# Editorial: The launch phase of *Bioengineering & Translational Medicine*

**DOI:** 10.1002/btm2.10140

**Published:** 2019-07-25

**Authors:** Samir Mitragotri

**Affiliations:** ^1^ Biologically Inspired Engineering Harvard University Boston Massachusetts

1

I am delighted to report on the exciting progress made by *Bioengineering & Translational Medicine* (BioTM) during its launch phase. The journal was formally announced in 2015 and the first issue was published in 2016. This issue marks the completion of the fourth year of our publication. BioTM was launched to feature exciting fundamental and translational research at the interface of Chemical Engineering and Medicine/Biological Sciences. The journal has had a particular focus on the translational aspects of bioengineering research.

Translation has undoubtedly become a key facet of scientific research in recent years. Engineering research has led to numerous scientific discoveries that have the potential to transform clinical medicine. However, converting these discoveries into actual useful clinical technologies is a major challenge. Several factors determine the likelihood of success of such translation including safety, manufacturability, regulatory hurdles, cost, and patient acceptance. These issues are not typically considered in fundamental academic research, yet they are increasingly becoming a key part of the DNA of academic research. Clinical translation also often requires concurrent commercial translation, which brings its own challenges and constraints.^1^ As we collectively advance our research and seek to increase its societal impact, these translational considerations must be catered to and adequately addressed. BioTM is a forum for discussing these issues and publishing manuscripts that provide new insights into understanding and overcoming these issues. Toward this goal, we have published several articles that provide a critical assessment of success and challenges in some of the key areas of interest including nanomedicine,^2^ gene therapy^3^ responsive materials and biotechnology.^4^, ^5^ In terms of scientific topics, BioTM has focused on subjects where Engineering, Chemical Engineering in particular, has made a strong fundamental and translational impact on the field. These topics include drug delivery,^6^, ^7^, ^8^, ^9^, ^10^ Nucleic acid delivery,^3^, ^11^, ^12^, ^13^, ^14^ Regenerative Medicine,^15^, ^16^, ^17^ Biotechnology,^4^, ^18^ Nanomedicine,^19^, ^20^, ^21^, ^22^, ^23^ Biomedical Devices,^24^ and immunotherapy.^25^, ^26^ We look forward to continue building our strengths in these fields and expand the list to include additional key areas.

I want to take this opportunity to thank our supporters during the launch phase. We are grateful for the strong support and participation of our editorial board members. Our stellar advisory board represents 32 memberships of National Academies of Engineering, Medicine and Science. Our board members have provided the guidance and the vision to the journal, and have published their own research in BioTM. To celebrate our stellar advisory board, we started a feature to introduce them in each issue. To date, we have written features about Profs. Nicholas Peppas, David Tirrell, Matthew Tirrell, Mark Davis, Paula Hammond, Frank Doyle, R.A. Mashelkar and Kristi Anseth. These editorial profiles can be found on our website. We look forward to showcasing the biographies of additional board members in coming issues.

I want to especially thank the AIChE and SBE community for the strong support to the journal, especially during the launch of a new conference in Bioengineering and Translational Medicine that accompanies BioTM journal. The first conference was chaired by Profs. Mark Prausnitz and Ravi Kane in San Francisco. The following conferences were chaired by Prof. Ali Khademhosseini and Efrosini Kokkoli (2017, Minneapolis), Profs. Rebecca Carrier and Stelios Andreadis (2018, Boston), and Profs. Ashutosh Chilkoti and Joel Collier (2019, Raleigh). Two special issues were also published from the 2017 edition of this conference. (https://aiche.onlinelibrary.wiley.com/toc/23806761/2018/3/2 and https://aiche.onlinelibrary.wiley.com/toc/23806761/2018/3/3) (edited by Prof. Kannan Rangaramajunam).

We are also pleased with the enthusiastic participation of the young generation of researchers, especially from the AIChE community, in the operation of the journal in various capacities including guest editors, authors, and reviewers. In particular, I want to thank Prof. Aaron Anselmo, who started our feature BioTM Buzz which highlights select articles from each issue along with some additional articles published elsewhere in the literature. Follow BioTM_Buzz on Twitter for updates on our publications. We are also thankful to Profs. Kaushal Rege, Kannan Rangaramajunam, Paolo Decuzzi, Zhen Gu, Kathryn Whitehead, Pankaj Karande, Josue Snitzman, Millicent Sullivan for guest‐editing special issues. Without the enthusiastic participation of these committed individuals, we would not have gotten off to such a great start. We also thank Arthur Baulch for excellent management of the operation, Cynthia Mascone for leadership in publication policies, and Mia Ricci and the Wiley team for publication strategies.

As we start our fifth year, I look forward to many more exciting developments. BioTM was recently selected for coverage in Clarivate Analytics and is now fully indexed in Web of Science, in addition to full indexing in Pub Med Central (PMC). The journal is now scheduled to receive its initial impact factor in 2020, which is an important milestone. I also look forward to the growth phase of the journal including expansion of covered topics, broader participation of industry researchers in the journal and a more critical discussion/analysis of translational challenges in the field. I thank you all for your strong support to the journal to date and look forward to your continued support in coming years.
